# Immobilization of tris(2 pyridyl) methylamine in a PVC-Membrane Sensor and Characterization of the Membrane Properties

**DOI:** 10.1186/1752-153X-6-40

**Published:** 2012-05-07

**Authors:** Majid Rezayi, Lee Yook Heng, Anuar Kassim, Saeid Ahmadzadeh, Yadollah Abdollahi, Hossein Jahangirian

**Affiliations:** 1 School of Chemical Sciences and Food Technology, Faculty of Science and Technology, Universiti Kebangsaan Malaysia, Bangi, Selangor DE, 43600, Malaysia; 2 Food Science and Technology Research Institute, ACECR Mashhad Branch, Mashhad, Iran; 3 Department of Chemistry, Faculty of Sciences, Universiti Putra Malaysia, Serdang, Selangor, 43400, Malaysia; 4 Advanced Materials and Nanotechnology Laboratory, Institute of Advanced Technology, Universiti Putra Malaysia, Serdang, Selangor DE, 43400, Malaysia

## Abstract

**Background:**

Due to the increasing industrial use of titanium compounds, its determination is the subject of considerable efforts. The ionophore or membrane active recognition is the most important component of any polymeric membrane sensor. The sensor’s response depends on the ionophore and bonding between the ionophore and the target ion. Ionophores with molecule-sized dimensions containing cavities or semi-cavities can surround the target ion. The bond between the ionophore and target ion gives different selectivity and sensitivity toward the other ions. Therefore, ionophores with different binding strengths can be used in the sensor.

**Results:**

In the present work, poly (vinyl chloride) (PVC) based membrane incorporating tris (2 pyridyl) methylamine (tpm) as an ionophore has been prepared and explored as a titanium(III) selective sensor.

**Conclusions:**

The strengths of the ion–ionophore (Ti(OH)^2+^-tpm) interactions and the role of ionophore on membrane were tested by various techniques such as elemental analysis, UV–vis, Fourier transform infrared (FTIR) spectroscopy, scanning electron microscopy (SEM), and powder X-ray diffraction (XRD). All data approved the successful incorporation of organic group via covalent bond.

## Background

**Figure 1:**
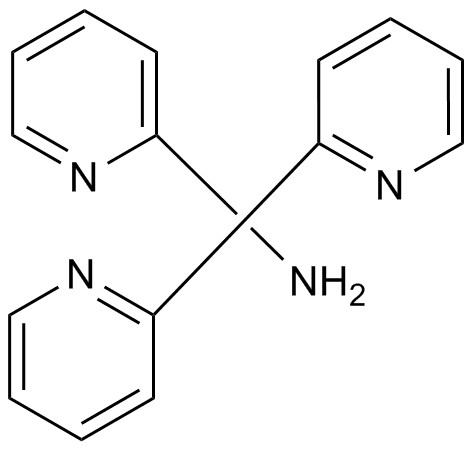
Chemical structure of tris(2 pyridyl) methylamine.

Tripodal molecules are a group of compounds with three arms of donor groups. As a flexible virtue group like Sexipyridines, this new type of ligand can form complexes with metal ions. The first new tripodal compound was reported in 2000 by Berrocal et al. with sulfate recognition properties for anion-selective electrodes [1]. Yan et al. synthesized 1,1,1-tris(N-ethyl-N-phenylamino-carboxylmethoxymethyl) propane and used it as an ionophore in PVC membrane electrode for the analysis of alkali and alkaline earth metal cations [2]. Kim et al. published a paper on new C-3 symmetric, tripodal trifluoroacetophenone derivatives as anion ionophores [3]. Several studies have shown that the tripodal ligands can be categorized by two main groups: flexible tripodal ligands and rigid tripodal ligands. The presence of donor groups in the tripodal ligand leads to complexation of the ligand and metal complexes with a good formation constant [4]. The donor groups in this branch of tripodal ligands are usually donor soft groups that are connected to nitrogen atoms. This makes the molecule resemble a flexible tripod. The chemical structure of tris (2 pyridyl) methylamine (tpm) as an ionophore used in this study is shown in Figure 1.

The tpm ionophore as a basic component is responsible for the potential response of ion selective electrodes (ISEs) and has been used to prepare the membrane. According to the conductometric method, the tpm ionophore forms 1:1 complexes with titanium (III) cations [5-12].

**Figure 2:**
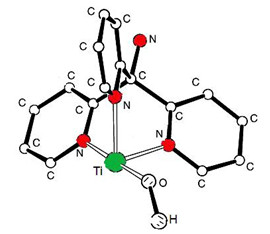
**Optimal conformation of tpm-Ti(O H)**^**2+**^**complex.**

The optimized structures of the tpm-Ti(OH)^2+^ complex are shown in Figure 2[13]. In the complexed forms, the benzo groups are not coplanar due to the OH group in the Ti(OH)^2+^ cation, which is far enough from the N and O groups in tpm to minimise the possible intermolecular repulsive force [12]. In the tpm-Ti(OH)^2+^ complex, the amine hydrogen atoms are refined freely. The N atoms in the Pyridines donor groups are in suitable proximity of the central Ti cation for proper binding interactions [14-17].

In this work, the new type of membrane based on tpm ionophore as a tripodal ligand to determine the titanium (III) cation was presented. The properties of the tripodal ionophore immobilized in the PVC matrix in comparison to the free tripodal ionophore system were investigated. For this study, poly(vinyl chloride) (PVC) based membrane incorporating tpm as an ionophore was prepared and explored as titanium(III) selective sensor. The strengths of the ion–ionophore (Ti(OH)_2_^+^-tpm) interactions and the role of ionophore on membrane were also studied via UV–vis, Fourier transform infrared spectroscopy (FT-IR), powder X-ray diffraction (XRD) and scanning electron microscopy (SEM).

## Results

### UV–Vis studies

**Figure 3:**
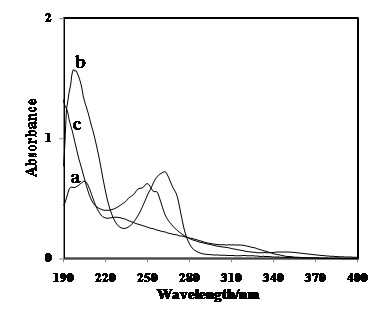
**(a) UV–vis absorption spectra of acetonitrile solutions of 1.0 × 10**^**-4**^ **M TiCl**_**3**_**; (b) 1.0 × 10**^**-4**^ **M tpm in the absence of TiCl**_**3**_**; (c) tpm 1.0 × 10**^**-4**^ **M treated with 1.0 × 10**^**-4**^ **M TiCl**_**3**_**solution**.

The UV–vis was used to investigate the interaction between the tpm ionophore and the Ti(OH)^2+^ cation. In order to study the selective interaction of the tpm as potential ionophore, the UV–vis spectra of the complex was obtained in the absence and presence of this cation in dry acetonitrile solution. According to the Hofmeister series (lyotropic series – favourable ability of the cations to complexation) for cations, significant interaction between tpm with Ti(OH) was expected [18]. As shown in Figures 3, the results support this.

### FT-IR studies

**Figure 4:**
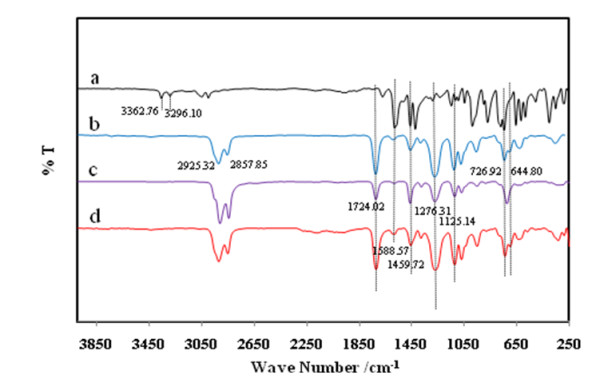
**The FT-IR spectra of the membrane PVC sensor based on tpm ionophore in different states: (a) pure tpm, (b) blank membrane, before (c), after (d) destocking in 1.0 × 10**^**-3**^ **M TiCl**_**3**_**solution for 24 hours**.

The influences of ionophores, titanium cation, internal solution, and life time of the proposed membrane sensors were investigated by FT-IR spectra. Figure 4 shows the characteristic peaks of FT-IR for the tpm membrane sensor in four steps (A) tpm ionophore, (B) blank membrane, before (C), after (D) destocking in 1.0 × 10^-3^ M TiCl_3_ solution for 24 hours.

### SEM studies

**Figure 5:**
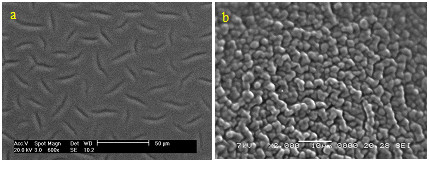
**Scanning electron microscopy pictures of the surfaces of the tpm-immobilized membranes (a) without ionophore, (b) with ionophore**.

Scanning electron microscopy (SEM) indicated that addition of the tpm ionophore into the PVC membrane produced a loose and preamble structure electrode surface (Figure 5).

### X-ray studies

**Figure 6:**
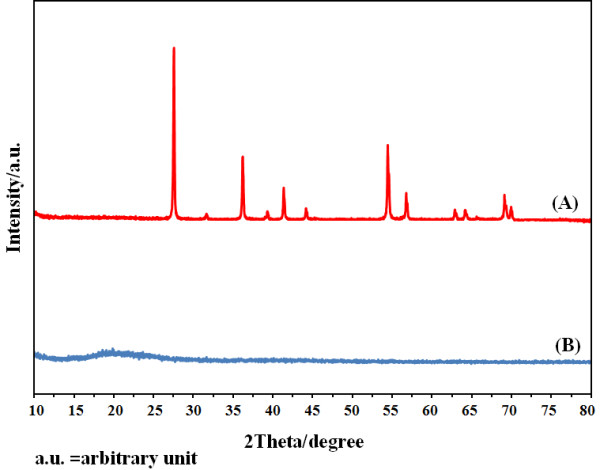
**X-ray diffraction patterns of (a) pure TiO**_**2**_**, (b) membrane based tpm ionophore, stocked in 1.0 × 10**^**-2**^ **M TiCl**_**3**_**solution**.

The X-ray diffraction (XRD) facilitates understanding of the physical properties of metals, polymeric materials, and other solids. When an X-ray beam strikes a crystal, a portion of this beam is scattered by the layer of atoms at the surface. If the distances between the scattering centers are of the same order of magnitude as the wavelength of the radiation, then interference takes place among the scattering rays, resulting in diffraction. Figure 6 shows the X-ray diffraction patterns of the optimized polymeric membrane based on the tpm ionophore after stocking 24 hours in 1.0 × 10^-2^ M TiCl_3_.

## Discussion

The preliminary result of interaction between the ionophore and metal ion was investigated by the UV–vis spectroscopy method. With UV–vis spectra, as illustrated in Figure 3, it was immediately obvious that the addition of ionophore (tpm) (with two absorption maxima at 199 and 262 nm) to an equilibrium solution of Ti(OH)^2+^ (with two absorption maxima at 196 and 205 nm) had resulted in a decrease of absorbance and shift to 190 and 250 nm. The substantial decreases in the absorbance at 190 and 250 nm after the contact of the titanium (III) cation solution with a tripodal ligand-containing phase suggested that the absorbing species had increased in size and axial coordination could have taken place. At the same time, the effects of other cations such as cadmium, lead, zinc, calcium and magnesium on the spectrum of the carrier were investigated and no detectable changes in the UV/Vis spectra were noted. These results revealed that the tpm ionophore had especial tendency to the Ti(OH)^2+^ ions respect with other common cations. The obtained results denoted that the mechanism of the response of the membrane sensor to Ti(OH)^2+^ was based on coordination of Ti(OH)^2+^ ion to tpm and its rapid exchanges with the solution containing Ti(OH)^2+^ ions.

The influences of ionophore, titanium cation of the proposed membrane sensors were investigated by FT-IR spectra. Figure 4 shows the characteristic peaks of FT-IR for the tpm membrane sensor in four steps (A) tpm ionophore, (B) blank membrane, before (C), after (D) destocking in 1.0 × 10^-3^ M TiCl_3_ solution for 24 hours. The presence of the Ti(OH)^2+^ cation caused a shift and an increase in the absorption intensity of the C-C stretching in the pyridine rings at 1459.72 and 1588.57 cm^-1^ (spectrum D) in the Ti(OH)^2+^-tpm complex and in the OH stretching at 3242.14 cm-1 (spectrum C) in Ti(OH)^2+^-tpm complex [19]. These displacements were attributed to pyridine C-N and O-H stretching band by Ti-N and Ti-O coordination [16]. Clearly, there was no peak related to the ionophore (C-C stretching in the pyridine rings) in the blank membranes (spectrum B). The presence of DOP as plasticizer in the membrane compositions based on tpm ionophore was confirmed by the strong absorption bands related to C – O stretching appearing at 1100–1300 cm^-1^ region and C = O stretching appearing at 1724 cm^-1^. As observed from all three FT-IR spectra (B, C and D spectrums), both plasticizers (DOP) and ionophores (tpm) showed the common peak of C – H sp^3^ stretching in 2857 cm^-1^ region. Besides, the common peak of = C – H sp^2^ stretching appeared at 2925 cm^-1^ indicating the presence of plasticizer (DOP) and lipophilic additives (KTK). It is noteworthy to mention that, due to a very low amount of lipophilic additive in the matrix of the membranes and high intensity of C = O, C = C absorption bands which overlapped with the C – Br stretchings, it was not possible to deduce the presence of lipophilic additive individually from the presented FTIR spectra.

With the development of new surface analysis techniques such as scanning electron microscopy (SEM), it is possible to image the surfaces of some non-conducting samples like selective PVC membrane to distinguish their surface characteristics such as fouling and swelling. In order to investigate the surface morphology of titanium (III) cation selective membranes, SEM studies were carried out at different magnifications.

Figure 5 shows the surface morphology of the fabricated membrane as investigated by SEM. The PVC-membrane without the tpm ionophore exhibited a physically tight structure, as illustrated in Figure 5, while the membranes with the tpm ionophore exhibited a surface with a loose and permeable structure that included channels to diffuse the Ti(III) cations.

As illustrated in Figure 6, the XRD patterns of the fabricated membrane based on tpm (pattern B) did not indicate any crystalline character. These observations evidence the amorphous structures of the fabricated membranes without any crystalline structure due to the presence of TiO_2_ (pattern A). These results showed that the dominant titanium species in the crystal structure of titanium chloride (III) to be in the Ti(OH)^2+^ form.

## Conclusions

In this study, tpm was applied as a sensing element to determine the titanium (III) cations in PVC membrane sensor. Membrane based on the tpm ionophore was prepared with a composition of 5.0:61.5:33.0:0.5 (mg) tpm:DOP:PVC:KTK. The selectivity of the tpm ionophore toward the titanium (III) cation was quite good compared to those of the other studied cations. Hence, the proposed membrane sensor shows excellent selectivity for Ti(OH)^2+^. The results demonstrate that the application of tpm in the polymeric membrane phase as a selective ionophore created cation-sensor for the quantification of titanium (III) cations. Accordingly, we used this ligand as a sensing element in the membrane to fabricate the ion selective electrode [20,21].

## Methods

### Reagents

Deionized bi-distilled water was used to prepare the solutions. tris(2 pyridyl) methylamine (tpm, synthesized and purified according to the work of Arnold et al. 2001). A TiCl_3_ solution of about 15% was purchased from Merck. Polyvinyl chloride powder of high molecular weight (PVC, Fluka) and tetrahydrofurane (THF, Fluka) were used without further purification. Dioctylphthalate (DOP, Merck), potassium tetrakis (4-chlorophenyl) borate (KTK, Fluka) as a sensor plasticizer, lipophilic anionic additive were used, respectively.

### Apparatus

The Fourier transform infrared spectroscopy (FT-IR) spectra of the samples was recorded via a Perkin Elmer Fourier Transform Infrared spectrophotometer (model spectrum 100 series) using a KBr disc. For UV–vis spectroscopy, a Perkin-Elmer Lambda spectrophotometer 1650 pc (SHIMADZU) was used. The scanning electron microscopy (SEM) techniques using a Jeol scanning electron microscope (Model 1455 LEO) for immobilization of ionophore was used. X-ray diffraction (XRD) scans were taken on a SHIMADZU XRD-6000 Lab X wide-angle diffractometer to characterize the membrane samples.

### Experimental procedures

After preparation of membrane, it was conditioned in a 1.0 × 10^-3^ mol L^-1^ TiCl_3_ solution for about 24 h. FT-IR was used to characterize the sample composition of the membrane in this study. The absorption (or transmission) spectra of the molecular species samples was recorded in the 400 to 4000 cm^-1^ wave number range by a Perkin Elmer Fourier Transform Infrared spectrophotometer (spectrum 100 series) using the KBr pellet technique. UV–vis spectroscopy was used for the Ti(OH)^2+^-tpm complexation study. Three sample solutions were prepared (1 × 10^-4^ M solution of tpm in the absence and the presence of 1 × 10^-4^ M of TiCl_3_) in a binary mixture of acetonitrile-water. Then, the absorption spectra of solutions was recorded for each sample in the range of 190 to 400 nm with a Perkin Elmer model SHIMADZU, 1650 pc UV–vis spectrophotometer. For the SEM study, the sample holders were cleaned and then dried with acetone solvent. The sample membranes were then fixed onto the holders and coated with gold in the vacuum chamber to increase the conductance of their surfaces. These samples were used to study the morphology of the membrane surfaces by SEM use.

### Analytical method for preparing membrane sensor

Membrane solutions were prepared by thoroughly dissolving 5.0 mg of the tris(2 pyridyl) methylamine (tpm), 61.5 mg of di-n-octyl phthalate (DOP), 33.0 mg of PVC and 0.5 mg of potassium tetrakis (4-chlorophenyl) borate (KTK) in 3 ml of fresh THF. The resulting solution evaporated slowly until an oily mixture was obtained. A Pyrex tube (3 mm o.d) was dipped into the mixture for about 10 s, so that a nontransparent membrane of 0.3 mm thickness formed. The tube was then removed from the mixture and kept at room temperature for 24 h.

## Competing interests

The authors declare that they have no competing interests.

## Authors' contributions

These authors contributed equally to this work. All authors read and approved the final manuscript.
